# Management and prevention of chronic obstructive pulmonary disease exacerbations: a state of the art review

**DOI:** 10.1186/1741-7015-7-40

**Published:** 2009-08-07

**Authors:** John R Hurst, Jadwiga A Wedzicha

**Affiliations:** 1 Academic Unit of Respiratory Medicine, Royal Free Campus, UCL Medical School, London, UK

## Abstract

Exacerbations of chronic obstructive pulmonary disease (COPD) are important events in the natural history of this prevalent and devastating condition. This review provides a concise, state of the art summary on prevention and management of exacerbations. Considerable new data underpins evidence in support of many preventative interventions, pharmacological and non-pharmacological, that are now available. Challenges remain in developing new approaches, and delivering those that already exist to the right patient at the right time. Management of an exacerbation remains stepwise according to clinical severity, but there is now additional focus on addressing comorbidities and taking the opportunity at acute events to optimise preventative strategies for the future. Ultimately, exacerbations are heterogeneous events in a heterogeneous disease, and an individualised approach is paramount.

## Background

Affecting 10% of the population over the age of 40 years [1], the burden of chronic obstructive pulmonary disease (COPD) has reached epidemic proportions. COPD is defined by the presence of poorly reversible airflow obstruction and an abnormal inflammatory response in the lung to noxious particles or gases [2]. Punctuating the decline in lung function are acute deteriorations in respiratory health, termed exacerbations. As discussed further below, exacerbations are important events in the natural history of COPD. This review provides a critical summary of present exacerbation therapies and aims to update the reader on recent developments in the treatment and prevention of exacerbations in COPD.

## The biology and importance of COPD exacerbations

Exacerbations are key events in COPD, defined by the presence of worsening symptoms but also often associated with concurrent deteriorations in pulmonary function and increases in both local and systemic inflammation [3]. Exacerbations are caused by those insults that increase airway inflammation, principally episodes of bronchial infection, but also pollutants or stimuli that directly affect expiratory flow limitation [3].

Although it is generally true that exacerbations become more frequent as the severity of the underlying COPD increases [4], there are large differences in exacerbation incidence rates ('exacerbation frequency') between individual patients, and patients susceptible to more frequent exacerbations ('frequent exacerbators') appear to be a distinct phenotype susceptible to a more rapid decline in lung function [5,6], poorer quality of life [7] and increased mortality [8]. They are therefore a particularly important group to target for the exacerbation prevention strategies that are described below. There is emerging evidence to suggest that a proportion of patients with milder COPD may be susceptible to frequent exacerbations [9].

Exacerbations are very heterogeneous events ranging from no more than troublesome increases in respiratory symptoms to life-threatening episodes of respiratory failure. A typical community-treated exacerbation in a patient with moderately severe COPD has a short prodrome and a median symptom duration of 7 days, though some are considerably longer and a proportion of patients may never completely return to their pre-exacerbation baseline [10]. Exacerbations in more severe disease commonly require hospitalisation, and they are a much more common cause of emergency hospital admission than, for example, exacerbations of asthma [4]. Therefore, in addition to significant personal detriment to the patient, exacerbations of COPD demand a considerable portion of healthcare expenditure. Moreover, with an ageing population and relatively greater reductions in mortality from other prevalent diseases (notably cardiovascular disease) the burden of exacerbations continues to rise [1].

## Exacerbation diagnosis and treatment

Exacerbations of COPD remain a clinical diagnosis of exclusion, and it is necessary to consider (and, where appropriate, rule out) other causes of increased breathlessness in patients with COPD presenting with symptom deteriorations [11]. Conditions mimicking exacerbations include pneumonia, pneumothorax, pulmonary embolus and cardiac failure. Diagnosis of exacerbation therefore requires appropriate clinical assessment and may need further investigation with, for example, chest radiography.

The principles of exacerbation therapy have not changed greatly since the widespread introduction in the 1990s of non-invasive ventilation in preference to doxapram for the treatment of hypercapnoea at exacerbation of COPD. Therapies at exacerbation may be divided into those thought to have a disease-modifying effect, and those aiming to support respiratory function until disease-modifying therapies have had sufficient time to act.

**Figure 1:**
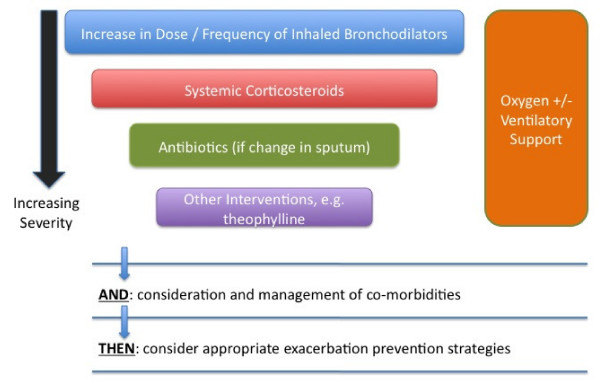
**General scheme for management of a chronic obstructive pulmonary disease (COPD) exacerbation**. Therapy is added stepwise according to the severity of the presentation. Mild exacerbations may respond to an increase in the dose and/or frequency of inhaled bronchodilators alone. Exacerbations not responding to this require systemic corticosteroids, with the addition of antibiotics if there has been a change in the character of expectorated sputum. Additional interventions such as theophylline may be required where the clinical response is still incomplete. Oxygen and/or ventilatory support may be necessary at any stage in the presence of new or established respiratory failure. Exacerbation management should also include an assessment and management of comorbidities, and the opportunity should be taken to optimise long-term therapies to reduce the risk of future exacerbations.

Exacerbation therapy is administered stepwise according to the clinical severity of presentation, and a general scheme is presented in Figure 1. It is important to note that disentangling the concept of exacerbation severity from the severity of the underlying COPD is almost impossible: a patient needing intubation and ventilation at exacerbation may have milder disease with a greater acute deterioration, or more severe underlying disease and a trivial insult.

The mainstay of therapy at exacerbation remains an increase in the dose and frequency of short-acting β_2 _agonist and anticholinergic bronchodilators, and systemic corticosteroids for exacerbations not responding to this intervention alone. Antibiotics are generally added for exacerbations associated with a change in the characteristics of expectorated sputum. Oxygen with or without ventilatory support is necessary in the presence of respiratory failure, and often indicates the need for hospitalisation. Theophyllines are sometimes added in patients responding poorly to other therapies. Many national and international guidelines exist [12,13].

Reviewing data from the original trials of these commonly prescribed therapies challenges our rationale for their use. Many exacerbations in the seminal study of antibiotics versus placebo improved in the placebo arm anyway [14], and the value of antibiotics has recently been further questioned in a systematic review in this journal [15]. There certainly appears to be no additional benefit from antibiotic courses lasting longer than 5 days compared to shorter regimes [16]. The major benefit of systemic corticosteroids is in increasing the rate of recovery of lung function, rather than more important endpoints such as mortality [17]. Indeed, there is evidence of relative corticosteroid resistance in COPD [18]. Whilst acknowledging the methodological limitations of such approaches, meta-analyses are available reporting summarised benefits at exacerbation for short-acting bronchodilators [19], systemic corticosteroids [20], antibiotics [21], theophyllines [22] and non-invasive ventilation (NIV) [23]. NIV has now all but replaced respiratory stimulants such as doxapram in the management of exacerbations with acute hypercapnoeic respiratory failure, and meta-analyses of NIV have documented a mortality benefit from such therapy over standard care [23]. However, NIV is not a replacement for endotracheal intubation and ventilation where this is necessary through severity or contraindication to NIV. Whilst the decision to intubate and ventilate a patient with COPD can be complex, outcomes in COPD are no worse than in patients with respiratory failure due to other causes [24] and any remaining therapeutic nihilism in this area should be challenged.

There is continuing interest in novel models of care including 'hospital at home'. Most data suggest that for selected patients such schemes are safe, and often preferred by patients [25], but that they are not necessarily more cost effective than traditional approaches.

Whilst pulmonary rehabilitation has an important role in preventing hospitalisations in COPD (discussed below), the concept of 'early rehabilitation' commenced during an acute exacerbation has not been definitively associated with improved outcomes. These results from a recent randomised controlled trial [26] contrast with those from an earlier meta-analysis of six smaller studies [27].

Intriguingly, further new data has suggested that treatment provided at a first exacerbation may affect the timing of second events. The addition of antibiotics to steroids, for example, has been associated with a reduced risk of subsequent recurrent exacerbation [28]. Indeed, it is now recognised that exacerbations are not random events, but rather cluster together in time such that in the period immediately following a first exacerbation there is increased risk of a second [29].

Future developments might be expected to include the validation of biomarkers to provide information on exacerbation aetiology and novel drugs, particularly antiviral agents and alternative anti-inflammatory agents to corticosteroids. Use of procalcitonin to inform on the likelihood of bacterial aetiology has been shown to safely reduce the use of antibiotics at exacerbation [30], but this concept is now complicated by the data referred to above suggesting that antibiotic treatment at one exacerbation may affect the timing of subsequent events [28]. It is also important to remark that the presence of bacteria in sputum at exacerbation does not imply that the organism is causative, and sputum bacterial colonisation is a frequent consequence of advancing COPD severity [31].

A final aspect of exacerbation therapy is data demonstrating that access to early treatment speeds exacerbation recovery [32] and this has led to many patients receiving 'emergency' courses of treatment to keep at home. However, data to specifically support self-management strategies (which requires self-diagnosis) are absent [33,34].

## Exacerbation prevention

In contrast to treatment of acute events, recent years have seen publication of landmark trials in COPD which inform on exacerbation prevention and we now have a wide range of options available, both pharmacological and non-pharmacological, to reduce exacerbation frequency. These are listed in Table 1, which also includes interventions known to reduce COPD hospitalisation (a closely related concept). Perhaps the greatest remaining challenge is to establish which patient will benefit from which combination of approaches, though there is still the need for novel interventions as current approaches are incompletely effective.

**Table 1:** Interventions known to reduce exacerbation frequency or frequency of hospitalisation in chronic obstructive pulmonary disease (COPD)

Pharmacological	Non-pharmacological
Long-acting β_2 _agonists	Pulmonary rehabilitation
Long-acting anticholinergics	Lung volume reduction surgery
Inhaled corticosteroids	Long-term oxygen therapy
Mucolytics	Domiciliary non-invasive ventilation
Erythromycin (macrolide)	Influenza vaccination

## Recent trials

Although it has been known for some time that the three main classes of inhaled medication in COPD (corticosteroids, and long-acting anticholinergic and β_2 _agonist bronchodilatiors) all reduce exacerbation frequency, there is compelling new data with regard to these drugs.

The TORCH trial (for 'TOwards a Revolution in COPD Health') [35] was published in 2007 and compared placebo, salmeterol, fluticasone and salmeterol-fluticasone (SFC) against a primary end point of all cause mortality. A total of 6,112 patients formed the efficacy population and the study duration was 3 years. Mortality data was carefully collected. The study supports the concept that combination treatment with SFC may reduce mortality, but at *P *= 0.052 this just failed to achieve conventional statistical significance. Secondary outcomes included exacerbations and, supporting multiple earlier studies, there was evidence that all three active arms reduced exacerbations compared to placebo, with combination (SFC) treatment significantly better than either component alone. The annual rate of moderate and severe exacerbations in the placebo group was 1.13 per year, compared to 0.97 for salmeterol, 0.93 for fluticasone and 0.85 in patients receiving SFC. Like the subsequent trials described below, there was a significant preferential drop out rate from the placebo arm, complicating analysis, but which also represents a useful efficacy signal.

The Understanding Potential Long-term Impacts on Function with Tiotropium (UPLIFT) trial [36] was published in 2008 and compared the addition of tiotropium or placebo to current therapies with a primary end point of lung function decline. A total of 5,993 patients were randomised and the study duration was 4 years. The primary end point was negative, and once again the secondary endpoints included exacerbations. The addition of tiotropium resulted in a significant reduction in exacerbation frequency (0.73 per year tiotropium vs. 0.85 per year placebo, *P *< 0.001), which is important given that many patients were already taking combined inhaled corticosteroids with long-acting β_2 _agonists.

The latter 'triple combination' approach is common in advanced COPD, and the only study to specifically examine this to date has been the Canadian OPTIMAL study [37]. This randomised 449 patients on tiotropium to additional placebo, salmeterol or SFC and had a 1-year duration. There was not a reduction in the primary endpoint of exacerbations in patients on tiotropium plus other therapy compared to tiotropium alone. These results are in keeping with a recent network meta-analysis published in this journal [38]. However, hospitalisations due to severe exacerbations were reduced in the group receiving tiotropium and SFC compared to tiotropium alone. Once again, patients on placebo were more likely to drop out. These data therefore question the 'triple combination' approach, at least with respect to the prevention of exacerbations and raise the concerning possibility of a ceiling effect for exacerbation preventative strategies.

The only head-to-head comparison of tiotropium versus SFC has been the Investigating New Standards for Prophylaxis In Reduction of Exacerbations (INSPIRE) study [39]. This randomised 1,323 patients to salmeterol-fluticasone or tiotropium over 2 years. Both treatments, as expected, reduced exacerbation frequency, and to a similar degree (to 1.28 per year for SFC and 1.32 per year for tiotropium, with a ratio of rates of 0.97 (95% CI 0.84 to 1.12)). Intriguingly however, the remaining exacerbations appeared to differ in phenotype depending on which drug the patient was taking: patients on SFC were more likely to need antibiotics at exacerbation whilst patients on tiotropium were more likely to require steroids. Which patients benefit most from which drug remains to be clarified. Perhaps a more important decision, which also remains to be adequately addressed, is which strategy to employ first in patients with milder disease.

## Other strategies

Inhaled corticosteroids in combination with long-acting β_2 _agonists, and long-acting anticholinergics are the principal pharmacological approaches to exacerbation reduction in COPD at present. Whilst combination corticosteroid and long-acting β_2 _agonist therapy is recommended in both asthma and COPD, it is interesting to note that it is the inhaled corticosteroid that has had safety concerns in COPD, compared to the long-acting β_2 _agonist component in asthma [40]. There is also evidence that mucolytics such as carbocisteine may be effective, especially in patients not taking inhaled corticosteroids [41]. Macrolide antibiotics have additional anti-inflammatory action and a recent single-centre, double-blind randomised trial of erythromycin 250 mg twice a day over 1 year reported a 35% reduction in exacerbations [42]. Further confirmatory trials are ongoing. Other trials of prophylactic antibiotics are generally older [43], and there are ongoing concerns about the development of antibiotic resistance. Nevertheless, new strategies for the use of antibiotics are being studied such as intermittent or 'pulsed' therapy. As exacerbations are associated with additional inflammation, the effect of corticosteroids and macrolides in reducing exacerbations is perhaps to be expected. However, this would not explain the beneficial effect of 'pure bronchodilator' drugs such as tiotropium. It seems likely that a reduction in hyperinflation can also reduce exacerbation frequency (though tiotropium also has effects on mucus rheology).

New developments include ultra-long-acting (once daily) β_2 _agonists and corticosteroids, and bronchodilators which possess both antimuscarinic and β_2 _agonist activity. New classes of drugs are in development too, though none have yet been demonstrated to have an effect on exacerbation frequency.

Depression and anxiety are common in COPD [44], and likely also affect symptom perception and therefore exacerbation presentation. It is therefore important to detect and treat comorbid psychological conditions in COPD, though a specific effect on exacerbation reduction has not been documented.

Non-pharmacological approaches include influenza vaccination [45] and the recognised benefit in reducing hospitalisation of group, multiprofessional exercise and educational programmes (pulmonary rehabilitation) [46]. These programmes appear greater than the sum of their parts, and benefit from peer support. The finding that lung volume reduction surgery (LVRS; an approach in severe disease with heterogeneous distribution) reduced exacerbation frequency was perhaps unexpected [47] and this may also reflect the benefits of reducing hyperinflation. Criteria for long-term oxygen therapy (LTOT) are well established, and LTOT is one of the few interventions proven to have a mortality benefit in COPD [48,49]. Whilst a specific effect of oxygen on reducing exacerbations has not been demonstrated, there is evidence that underuse of LTOT where indicated results in increased hospital admissions [50]. A recent study has suggested that domiciliary NIV for COPD patients with hypercapnoeic respiratory failure may improve survival [51], but robust data in this field with regard to exacerbations also remain to be produced.

## Conclusion

Exacerbations are important events in COPD. Management of the acute event remains dependent on inhaled short-acting bronchodilators, oral corticosteroids and antibiotics, with or without oxygen and ventilatory support. Comorbidities should be addressed. Given the importance of these events, effective care should also include deployment of strategies, both pharmacological and non-pharmacological, to reduce future exacerbations. Although new approaches are in development, a major current challenge is to understand which strategies to use in which patients at which stage of their disease. COPD exacerbations are heterogeneous events occurring in a heterogeneous disease and there is no 'one size fits all' approach to COPD exacerbation management and prevention.

## Abbreviations

COPD: chronic obstructive pulmonary disease; LTOT: long-term oxygen therapy; LVRS: lung volume reduction surgery; NIV: non-invasive ventilation; SFC: salmeterol-fluticasone.

## Competing interests

JRH has received honoraria for attending advisory boards from Astra-Zeneca and Chiesi, and financial assistance to attend meetings from Astra-Zeneca, Boehringer-Ingelheim and GlaxoSmithKline. JAW has received honoraria for lectures or attending advisory boards from Astra-Zeneca, Bayer, Boehringer-Ingelheim, Chiesi, GlaxoSmithKline, Novartis and Pfizer and has current research grant support from Astra-Zeneca and GlaxoSmithKline.

## Authors' contributions

The first draft was written by JRH; the manuscript was then revised and edited by JRH and JAW.

## Pre-publication history

The pre-publication history for this paper can be accessed here:


